# Studies of the Suitability of Fowlpox as a Decontamination and Thermal Stability Simulant for Variola Major

**DOI:** 10.1155/2009/158749

**Published:** 2010-01-28

**Authors:** Amanda E. Chambers, Melissa M. Dixon, Steven P. Harvey

**Affiliations:** US Army Edgewood Chemical Biological Center, 5183 Blackhawk Road, Aberdeen Proving Ground, MD 21010, USA

## Abstract

Variola major, the causative agent of smallpox, has been eradicated from nature. However, stocks still exist; thus, there is a need for relevant decontamination studies, preferably with nonpathogenic simulants. Previous studies have shown a similarity in response of vaccinia virus and variola major to various decontaminants and thermal inactivation. This study compared vaccinia and fowlpox viruses under similar conditions, using disinfectants and temperatures for which variola major data already existed. Most disinfectants showed similar efficacy against vaccinia and fowlpox, suggesting the utility of fowlpox as a decontamination simulant. Inactivation kinetics studies showed that fowlpox behaved similarly to variola major when treated with 0.1% iodine and 5.7% polyethyleneglycol nonylphenyl ether, 0.025% sodium hypochlorite, 0.05% sodium hypochlorite, and 0.1% cetyltrimethylammonium chloride and 0.05% benzalkonium chloride, but differed in its response to 0.05% iodine and 0.3% polyethyleneglycol nonylphenyl ether and 40% ethanol. Thermal inactivation studies demonstrated that fowlpox is a suitable thermal simulant for variola major between 40°C and 55°C.

## 1. Introduction

Poxviruses are large, double-stranded DNA viruses that infect a wide variety of hosts, ranging fromcaterpillars to humans. They are the largest animal virus, with a brick-shape, ranging in size from 200 to 400 nm long and 170 to 200 nm wide. Due to their large size, poxviruses can be visualized with light microscopy, although the fine details of the virus remain obscure. Poxvirus genomes encode the genes necessary for replication and immune modulation, with replication occurring in the host cell's cytoplasm. The genome is large compared to most viruses, containing 130–300 kb, depending on the virus type. The DNA is A+T rich and linear. Unlike most DNA-containing viruses, poxviruses encode their own polymerase [1].

Poxviruses are classified into two categories: entomopox (insect infecting) and chordopox (vertebrate infecting). The chordopox viruses are further divided into eight categories, including orthopox, which includes variola major and vaccinia virus, and avipox, which includes fowlpox virus [2]. 

Variola major, the virus that causes the contagious disease smallpox, has been considered one of the most destructive viruses in history, affecting 1/10th of all humankind and killing 300 million people in the 20th century alone [3]. The disease was considered fully eradicated in 1980. However, although variola major no longer exists in any known animal reservoirs and nature, stocks are still kept in the United States and Russia. Smallpox has a 20–30% lethality rate in unvaccinated individuals [4], although analysis of historical data suggests that 77.6% of cases are still protected against severe disease 70 years after vaccination [5]. 

Vaccina virus, long considered the prototype of variola major, has been used as both a simulant and vaccine for variola major. They show remarkable sequence similarity; however, vaccinia virus does not cause disease in healthy individuals, possibly due to the fact that variola major virus contains complement inhibitors 100 times more potent than those of vaccinia virus [6]. However, vaccinia can still cause serious infection in humans and is therefore not an ideal simulant.

The purpose of this paper is to investigate whether fowlpox, also a member of the chordopox family, can be used as a simulant for variola major. Fowlpox virus naturally infects chicken and turkey; however, it is not infectious to humans. Fowlpox virus has a genome consisting of 260 genes. Of the 260 genes, 65 are conserved homologues of chordopox genes. When compared by Hexamer and Glimer computer programs, sixteen fowlpox virus genes have a closest match to variola major. These genes encode for transcription factors, RNA-polymerase subunits, DNA repair, protein modification, structural proteins, and immune evasion proteins. Seventeen fowlpox virus genes closely match those of vaccinia virus [7].

The fowlpox genome is approximately 70–100 kbp larger than chordopox genomes. The large genome is due to several factors. First, the fowlpox genome contains a diverse array of genes. Several genes encoding cellular function show homology with proteins of unknown function from yeast, bacteria, roundworm, plant, and human. Another gene shows similar homology to a protein found in yeast, human, tomato and fruit fly. Secondly, the fowlpox genome contains host range genes, limiting infection to members of the avian family. Thirdly, the virus is so large due to the presence of novel cellular homologues [7].

## 2. Materials and Methods

### 2.1. Viral Strains and Cell Lines

Vaccinia strain WR (VR-119) and Fowlpox strain FPV-MDVgBH (Merck's Disease vaccine strain) (VR-2330) were obtained from the American Type Culture Collection (ATCC, Manassas, VA).

BHK-21 (CCL-10), BSC-40 cells (CRL-2761) and UMNSAH/DF-1 cells (chicken embryo fibroblast cells) (CRL-12203) were also obtained from ATCC.

### 2.2. Viral Production and Plaque Assays for Vaccinia Virus

Vaccinia virus was propagated in BHK-21 monolayer cultures in Dulbecco's Modified Eagle's Medium (DMEM) containing 10% heat-inactivated fetal bovine serum (FBS) at 37°C. Infected cells were harvested and centrifuged at 3,210 × g at 4°C for 10 minutes. Pellets were resuspended in DMEM containing 10% FBS, freeze-thawed for three cycles, sonicated for four minutes on ice and then centrifuged at 3,210 × g at 4°C for 10 minutes. The supernatant was collected and titered in BSC-40 cells and served as the source of virus for all experiments described in this paper.

BSC-40 cells were plated at a density of 5.0 × 10^5^ cells per well in six well plates for vaccinia plaque assays. Cells were allowed to reach confluence overnight at 37°C. in DMEM containing 10% FBS. Following absorption, medium was removed and replaced with Minimal Essential Medium (MEM) containing 5% FBS and 1% SeaPlaque Agarose. Forty-eight hours after infection, plaques were visualized by staining with 0.01% crystal violet. The plaques were counted, and the results were reported in plaque forming units per milliliter (pfu/mL).

### 2.3. Viral Production and Plaque Assays for Fowlpox Virus

Fowlpox virus was propagated in UMNSAH/DF-1 monolayer cultures in DMEM containing 10% FBS at 39°C. Infected cells were harvested and centrifuged at 3,210 × g at 4°C for 10 minutes. Pellets were resuspended in DMEM containing 10% FBS, freeze-thawed for three cycles, sonicated for four minutes on ice, and centrifuged at 3,210 × g at 4°C for 10 minutes. The supernatant was collected and titered in UMNSAH/DF-1 cells and served as the source of virus for all experiments described in this paper.

UMNSAH/DF-1 cells were plated at a density of 1.0 × 10^6^ cells per well in six well plates for the fowlpox plaque assay. Cells were allowed to reach confluence overnight at 39°C in DMEM containing 10% FBS. Following absorption, media was removed and replaced with 50% MEM, 50% DMEM, 2.5% FBS, and 1% SeaPlaque Agarose. Five days postinfection, plaques were visualized by staining with 0.01% crystal violet. Plaques were counted, and the results were reported in pfu/mL.

### 2.4. Disinfection Studies

The following disinfectants were tested for their efficacy against vaccinia and fowlpox viruses: ethanol (70%), isopropyl alcohol (50%), sodium hypochlorite (0.5%), formaldehyde (30%), benzalkonium chloride (10%), a mixture of cetyltrimethylammonium chloride (6.67%) and benzalkonium chloride (3.33%), and a mixture of iodine (1.75%) and polyethyleneglycol nonylphenyl ether (10%). All disinfectants were prepared in distilled, deionized water and then filtered through a 0.25 *μ*m filter.

The following concentrations of disinfectants were used to determine the inactivation kinetics of vaccinia and fowlpox viruses: ethanol (40%), sodium hypochlorite (0.05% and 0.025%), a mixture of cetyltrimethylammonium chloride (0.1%) and benzalkonium chloride (0.05%), a mixture of iodine (0.1%) and polyethyleneglycol nonylphenyl ether (5.7%), and a mixture of iodine (0.05%) and polyethyleneglycol nonylphenyl ether (0.3%). All disinfectants were prepared in distilled, deionized water and then filtered through a 0.25 *μ*m filter.

The plaque assays described above for vaccinia and fowlpox viruses were followed to determine the activity of the disinfectants, with minor changes. Vaccinia and fowlpox viruses were diluted 1: 10 in sterile water (control) or disinfectant. To test the infectivity of the treated viruses, the virus was treated with disinfectants for 1, 3, 5, and 10 minutes. To determine the inactivation kinetics of the individual disinfectants, the viruses were treated for 15, 30, 45, 60, 90, 120, 150, and 180 seconds.

### 2.5. Thermal Stability Studies

In order to determine the thermal stability of Vaccinia and fowlpox viruses were diluted in 0.85% saline, pH 4.5, phosphate buffered saline (PBS), pH 7.4, 10% skim milk, and heart infusion broth as described by Hahon and Kozikowski [8] for their work with variola major. Solutions were equilibrated to 40°C, 45°C, 50°C, and 55°C and then seeded with virus, resulting in a 1: 100 dilution. Samples were removed at 0, 15, 30, 45, and 60 minutes and cooled on wet ice. The plaque assays described above were then performed.

### 2.6. Storage Studies

Aliquots of vaccinia and fowlpox viruses were stored at −80°C and 4°C for one week. After the one week storage period, the viruses were diluted 1: 100 in sterile PBS heated to 56°C as described by Hahon and Kozikowski [8]. Samples were incubated at 56°C for 0, 15, 30, 45, and 60 minutes. At the indicated timepoint, samples were removed and cooled on wet ice. The plaque assays described above were then performed.

### 2.7. Determination of Rate Constant and Virus Half-Life for Chemical Inactivation Studies

To determine the order of the reaction, the following graphs were produced. To determine a first-order reaction, graphs of the natural log of the virus concentration versus time were produced. A linear line with a negative slope indicated a first-order reaction. To determine a second-order reaction, graphs of 1/virus concentration versus time were produced. A linear line with a positive slope indicated a second-order reaction. 

The rate of reaction for the first-order reactions was determined using the following equation: *V*_*t*_/*V*_0_ = *e* ^−*kt*^, where *V*_*t*_ is the concentration of virus at time, *t*, and *V*_0_ is the concentration of virus at the zero timepoint, and *k* is the reaction rate. The rate of reaction for the second-order reactions was determined using the following equation: (1/*V*_*t*_) = (1/*V*_0_) + *k* *t*, where *V*_*t*_ is the concentration of virus at time, *t*, *V*_0_ is the concentration of virus at the zero timepoint, and *k* is the reaction rate.

The virus half-life (*t*_1/2_) for viruses showing a first-order reaction rate was determined using the following equation: *t*_1/2_ = ln  2/*k*, where *k* is the rate constant determined using the equations described above. The *t*_1/2_ for viruses showing a second-order reaction rate was determined using the following equation: *t*_1/2_ = 1/(*V*_0_ × *k*), where *V*_0_ is the concentration of virus at the zero timepoint. 

### 2.8. Determination of Rate Constant, Virus Half-Life, Δ*H* (Heat of Activation), and Δ*S* (Entropy of Activation) for Thermal Inactivation Studies

The rate constant and virus half-life for the thermal inactivation studies were calculated as described above. The calculations of Hahon and Kozikowski [8] were used to determine Δ*H* and Δ*S*. As shown in Figure 3, Δ*H* may be obtained by the Arrhenius plot of the natural logarithm of the *k* values versus the reciprocal of the absolute temperature (1/*T*). A straight line with the slope −Δ*H*/R is obtained, where *R* is the universal gas constant, from which the Δ*H* can be calculated from. The Δ*H* units were calories per mole. Hahon and Kozikowski [8] described using the Eyring equation to solve for Δ*S*. The Eyring equation is as follows: *k* = (*K* *T*/*h*) × *e*(−Δ*H*/*R* *T*) × *e*(Δ*S*/*R*), where *K* is Boltzmann's constant, *T* is the absolute temperature, *h* is Plank's constant, *R* is the universal gas constant, and *k* is the rate constant calculated for the reaction. The Δ*S* were calculated in Calories per mole per degree Celsius.

## 3. Results

### 3.1. Chemical Toxicity

Chemical toxicity assays were performed by incubating BSC-40 and UMNSAH/DF-1 cells with the highest dose of chemical for the viral absorption time (one hour for BSC-40 and two hours for UMNSAH/DF-1). Following incubation, the cell morphology was observed using a light microscope. Toxicity effects were noted to ensure that cells were healthy enough to produce viral protein, allowing the infection process to occur. Additionally, the determinations of toxicity effects were important to ensure that any cell death was due to viral infection and not the treatment chemical.

The ethanol, isopropyl alcohol, and sodium hypochlorite had no observable effects on either cell line. The formaldehyde and the surface-active detergents (benzalkonium chloride, cetyltrimethylammonium chloride, and a mixture of iodine and polyethyleneglycol nonylphenyl ether) had some toxic effects on cells at the highest two dilutions, but cells that recovered and grew had normal morphology.

### 3.2. Inactivation of Vaccinia and Fowlpox Using Full Strength Chemicals

All the chemicals tested disinfected both vaccinia virus and fowlpox virus (Tables 1 and 2). Disinfection occurred in one minute or less for all the chemicals tested. Positive controls were run for all samples in triplicate. For each chemical tested, triplicate test plates of untreated virus (positive control) were also cultured on cells. In order for the results of the chemical decontamination timepoint to be considered valid, the average positive control titer was within 1-log unit of the known virus titer. These results were identical to the results reported by Tanabe and Hotta, using vaccinia and variola major [9].

### 3.3. Inactivation Kinetics of Vaccinia and Fowlpox

**Figure 1:**
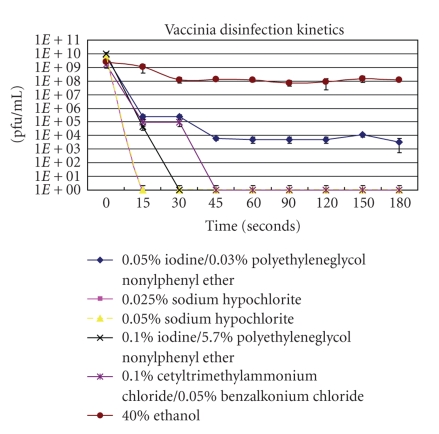
The inactivation kinetics of vaccinia virus following treatments with low concentrations of four different chemicals.

The four chemicals chosen for the inactivation kinetic studies were shown by Tanabe and Hotta to demonstrate high viracidal properties at minimum concentrations [9]. As shown in Figure 1, 0.05% and 0.025% sodium hypochlorite inactivated the vaccinia virus within 15 seconds of treatment. Within 30 and 45 seconds following treatment, 0.1% iodine and 5.7% polyethyleneglycol nonylphenyl ether and 0.1% cetyltrimethylammonium chloride and 0.05% benzalkonium chloride inactivated vaccinia virus, respectively. Two concentrations of chemicals were not high enough to result in complete viral inactivation. Following a three minute treatment with 40% ethanol, the vaccinia virus titer decreased by approximately 1-log pfu/mL. Although treatment for three minutes with 0.05% iodine and 0.3% polyethyleneglycol nonylphenyl ether did not result in complete inactivation, viral titers dropped by 5-log pfu/mL units.

The 40% ethanol inactivation kinetics in this study vary somewhat from the data reported by Tanabe and Hotta [9]. They reported that 40% ethanol inactivated variola and vaccinia within 1 minute. The simplest explanation of the differences may relate to purity of the viruses. Purified viruses are inactivated faster than nonpurified viruses. This is demonstrated by Tanabe and Hotta with variola major. Purified variola major virus is inactivated by 40% ethanol within 1 minute, while a nonpurified culture is inactivated within three minutes. Secondly, Tanabe and Hotta reported their data in percent reduction in plaque count. If the 40% ethanol inactivation kinetics described here were reported in percent reduction in plaque count, there would be a 95.4% reduction in plaques at the 60-second timepoint, which is the point at which Tanabe and Hotta describe a 100% reduction. 

**Figure 2:**
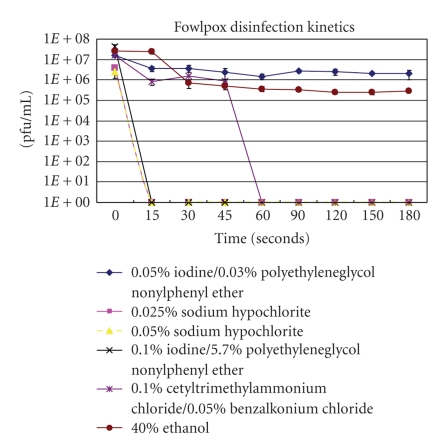
The inactivation kinetics of fowlpox virus following treatments with low concentrations of four different chemicals.

Fowlpox virus had an inactivation profile similar to that of vaccinia virus. Like vaccinia virus, 0.05% and 0.025% sodium hypochlorite inactivated the fowlpox virus within 15 seconds of treatment, as shown in Figure 2. Within 15 and 45 seconds following treatment, 0.1% iodine and 5.7% polyethyleneglycol nonylphenyl ether and 0.1% cetyltrimethylammonium chloride and 0.05% benzalkonium chloride inactivated fowlpox virus, respectively. The inactivation kinetic curves for 0.1% iodine and 5.7% polyethyleneglycol nonylphenyl ether and 0.1% cetyltrimethylammonium chloride and 0.05% benzalkonium chloride treated fowlpox are similar to the inactivation kinetic curves of vaccinia virus treated with those same chemicals. Like vaccinia virus, treatment for 3 minutes with 40% ethanol and 0.05% iodine and 0.3% polyethyleneglycol nonylphenyl ether did not result in complete inactivation of fowlpox virus. Following treatment of fowlpox virus with 40% ethanol for three minutes, the concentration of fowlpox virus dropped by approximately 2-log pfu/mL units. Fowlpox virus treated with 0.05% iodine and 0.3% polyethyleneglycol nonylphenyl ether had a decreased titer of approximately 1-log pfu/mL unit following a three-minute treatment. 

### 3.4. Data Analysis for Chemical Inactivation

Inactivation rate constants were calculated for vaccinia and fowlpox viruses treated with ethanol (40%), sodium hypochlorite (0.05% and 0.025%), a mixture of cetyltrimethylammonium chloride (0.1%) and benzalkonium chloride (0.05%), a mixture of iodine (0.1%) and polyethyleneglycol nonylphenyl ether (5.7%), and a mixture of iodine (0.05%) and polyethyleneglycol nonylphenyl ether (0.3%). The calculated rate constants for vaccinia and fowlpox viruses are shown in Table 3. 

Overall, vaccinia virus exhibited greater inactivation rate constants than fowlpox virus treated with the same chemicals. For both viruses, the order of the inactivation reaction when treated with 0.05% iodine and 0.3% polyethyleneglycol nonylphenyl ether was a second-order reaction. The viruses showed different reaction orders when treated with 40% ethanol. Vaccinia virus exhibited a first-order reaction, while fowlpox virus exhibited a second-order reaction. A first-order reaction was observed in both vaccinia and fowlpox virus treated with 0.05% and 0.025% sodium hypochlorite, a mixture of 0.1% cetyltrimethylammonium chloride and 0.05% benzalkonium chloride, and 0.1% iodine and 5.7% polyethyleneglycol nonylphenyl ether.

Vaccinia and fowlpox viruses treated with 0.1% iodine and 5.7% polyethyleneglycol nonylphenyl ether, 0.05% sodium hypochlorite, 0.1% sodium hypochlorite, and 0.1% cetyltrimethylammonium chloride and 0.05% benzalkonium chloride had a calculated half-life of less than 8.41 seconds (Table 3). The difference in virus half-life between the two viruses treated with the above chemicals was less than two seconds. When treated with 0.1% iodine and 5.7% polyethyleneglycol nonylphenyl ether, 0.05% sodium hypochlorite, 0.1% sodium hypochlorite, and 0.1% cetyltrimethylammonium chloride and 0.05% benzalkonium chloride, fowlpox had a slightly greater half-life than vaccinia virus by 1–2 seconds.

For two chemicals, the virus half-life of vaccinia and fowlpox virus differed greatly. Fowlpox virus treated with 0.05% iodine and 0.3% polyethyleneglycol nonylphenyl ether had a half-life 2.4 × 10^5^ times greater than the half-life of vaccinia virus treated with the same chemical. However, vaccinia virus treated with 40% ethanol had a virus half-life over 20 times greater than fowlpox virus treated with the same disinfectant.

### 3.5. Thermal Inactivation of Vaccinia and Fowlpox

#### 3.5.1. Stability Studies in 0.85% Saline

Vaccinia virus was completely inactivated in 0.85% saline when heated to 55°C. Approximately two-log reductions of viral titer were observed in the vaccinia diluted in 0.85% saline and heated at 40°C, 45°C and 50°C. According to the Arrhenius plot, the Δ*H* for vaccinia virus is 44,138 calories per mole in 0.85% saline (see Table 4). Unlike vaccinia virus, fowlpox virus was not completely inactivated in 0.85% saline when heated to 55°C. After one hour of heating at 55°C, fowlpox virus had a 2-log decrease in plaques. After heating for one hour at 40°C, 45°C, and 50°C, fowlpox virus titers did not decrease by even 1-log unit. The Δ*H* for fowlpox virus in 0.85% saline was 53,481 calories per mol in 0.85% saline (see Table 4). The entropy of activation (Δ*S*) for vaccinia virus in 0.85% saline ranged from 67.61 Cal/°C at 40°C to 65.85 Cal/°C at 55°C (see Table 5). The entropy of activation (Δ*S*) for fowlpox virus in 0.85% saline ranged from 71.70 Cal/°C at 40°C to 63.93 Cal/°C at 55°C (see Table 6).

#### 3.5.2. Stability Studies in PBS, pH 7.4

Vaccinia and fowlpox viruses behaved oppositely in PBS, pH 7.4 as compared to their behavior in 0.85% saline, pH 4.5. Unlike the vaccinia virus treated in 0.85% saline that was completely inactivated at 55°C, vaccinia virus in PBS, pH 7.4 is stable, decreasing by only 2-log units over the 60 minute incubation period. Vaccinia virus heated for one hour at 40°C, 45°C, and 50°C decreased by less than one-log unit. The calculated Δ*H* for vaccinia virus in PBS, pH 7.4, is 34,239 calories per mole (see Table 4). Unlike vaccinia virus, fowlpox virus was completely inactivated in PBS, pH 7.4 at 55°C. However, at 40°C, 45°C, and 50°C, fowlpox, like vaccinia, was stable, with titers decreasing by less than one-log unit over the hour long incubation period. The calculated Δ*H* for fowlpox virus in PBS, pH 7.4, is 35,868 calories per mole (see Table 4). The entropy of activation (Δ*S*) for vaccinia virus in PBS, pH 7.4, ranged from 69.08 Cal/°C at 40°C to 63.17 Cal/°C at 55°C (see Table 5). The entropy of activation (Δ*S*) for fowlpox virus in PBS, pH 7.4 ranged from 68.34 Cal/°C at 40°C to 62.45 Cal/°C at 55°C (see Table 6).

#### 3.5.3. Stability Studies in 10% Skim Milk

Vaccinia and fowlpox viruses behaved similarly when heated in a solution of 10% skim milk. Neither virus was completely inactivated at 55°C. After a one hour treatment at 55°C in 10% skim milk, vaccinia titers decreased by 4-log units and fowlpox virus decreased by 2-log units. After a one hour treatment in 10% skim milk at 50°C, vaccinia virus titers decreased by 2-log units, while fowlpox virus titers decreased by one-log unit. At the lower temperatures (40°C and 45°C) titers only dropped by one-log unit for both vaccinia and fowlpox viruses. The calculated Δ*H* for vaccinia virus in 10% skim milk is 44,741 calories per mole; while the calculated Δ*H* fowlpox virus in 10% skim milk is 22,378 calories per mole (see Table 4). The entropy of activation (Δ*S*) for vaccinia virus in skim milk ranged from 70.04 Cal/°C at 40°C to 62.82 Cal/°C at 55°C (see Table 5). The entropy of activation (Δ*S*) for fowlpox virus in skim milk ranged from 67.74 Cal/°C at 40°C to 65.12 Cal/°C at 55°C (see Table 6).

#### 3.5.4. Stability Studies in Heart Infusion Broth

Vaccina and fowlpox viruses also behaved similarly in heart infusion broth, both in their inactivation curves, but also in the fact that both viruses showed decreased aggregation at lower temperatures in this test liquid. Both viruses, especially noted in vaccinia as the time course progressed, plaque size dramatically decreased and titers actually increased between the 30- and 45- minute timepoints. At the 30- and 45-minute timepoints, both vaccinia and fowlpox plaques were approximately 10 times smaller than those observed during plaque assay optimization procedures, during which the virus was not treated with chemicals or heated. This could be indicative of decreased viral aggregation. However, by the 60-minute timepoint, titers had decreased to below or similar to the starting titer and plaque size returned to the normal sizes observed during plaque assay optimization.

Neither vaccinia virus nor fowlpox virus was completely inactivated at 55°C in brain heart infusion broth. Vaccinia titers decreased by 4-log units after a one hour incubation in heart infusion broth, while fowlpox virus titers decreased by 2-log units after a one hour incubation. At both 45°C and 50°C for both vaccinia and fowlpox viruses, virus titers did not decrease by one log-unit. The calculated Δ*H* for vaccinia virus in heart infusion broth is 95,204 calories per mole; while the calculated Δ*H* fowlpox virus in heart infusion broth is 65,229 calories per mole (see Table 4). The entropy of activation (Δ*S*) for vaccinia virus in heart infusion broth ranged from 63.12 Cal/°C at 40°C to 64.86 Cal/°C at 55°C (see Table 5). The entropy of activation (Δ*S*) for fowlpox virus in heart infusion broth ranged from 73.95 Cal/°C at 40°C to 63.55 Cal/°C at 55°C (see Table 6).

### 3.6. Storage Studies

**Figure 3:**
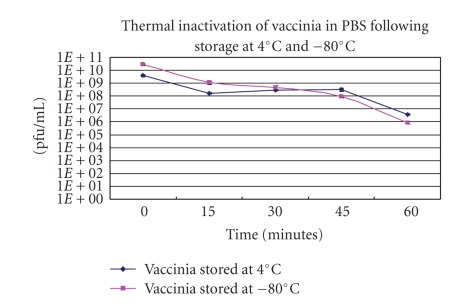
The inactivation of vaccinia virus stored at 4°C and −80°C for one week and inactivated at 56°C in PBS, pH 7.4.

Vaccinia and fowlpox viruses were stored for one week at either 4°C or −80°C, then diluted in PBS, pH 7.4, and inactivated at 56°C. Vaccinia virus was more stable than fowlpox virus under both storage conditions (see Figure 3). Following 60 minutes of heating at 56°C, vaccinia stored at 4°C showed a 3-log decrease in plaque forming units. Vaccinia stored at −80°C showed an approximately 4-log decrease in plaque forming units. Although titers decreased, viable virus still remained after the 60-minute incubation period. The reaction orders for both the virus stored at 4°C and −80°C were both first-order reactions.

**Figure 4:**
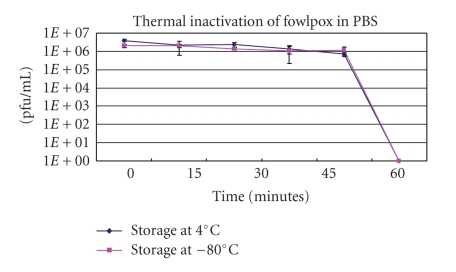
The inactivation of fowlpox virus stored at 4°C and −80°C for one week and inactivated at 56°C in PBS, pH 7.4.

Fowlpox varied significantly from vaccinia virus in its stability in PBS following different storage conditions (see Figure 4). Initially, fowlpox virus was heated at 56°C for 60 minutes; however, half-way through the incubation period, no viable fowlpox virus could be detected (data not shown). The experiment was repeated and instead of incubating the virus for 60 minutes at 56°C, the fowlpox was only incubated for 15 minutes at 56°C. When incubated for 15 minutes, inactivation kinetics could be determined. When the virus stored at 4°C was inactivated at 56°C, viral titers only dropped approximately one-log at the 15-minute timepoint, however, by the 30 minute timepoint, no active virus could be detected. When the virus stored at −80°C was inactivated at 56°C, viral titers declined ~0.2-log unit within the first 15 minutes, however like the sample stored at 4°C, after 30 minutes no active virus could be detected. Like the vaccinia virus, the fowlpox inactivation curves were both first-order reactions.

## 4. Conclusion

Vaccinia virus and variola major have significant sequence homologies. The viruses are so closely related that it has been hypothesized that vaccinia virus may have evolved from variola virus through continual passaging in cows and humans. Thus, vaccinia virus data common between this work and that of Tanabe and Hotta [9] and Hahon and Kozikowski [8] allow for an approximate comparison of the decontamination and thermal stability of fowlpox and variola major. 

Vaccinia virus is a well characterized virus that has been grown extensively in a variety of cell lines, from monkey and hamster kidney cells to chicken embryo fibroblasts. Fowlpox, due to the presence of host limiting genes in the viral genome, does not infect a wide variety of species like vaccinia. Based on literature searches, fowlpox virus is normally grown in primary chicken embryo fibroblast (CEF) cells. Due to the more difficult nature of maintaining primary cell lines, it has been demonstrated in this work that fowlpox can be grown in an immortalized CEF line, the UMNSAH/DF-1 cell line. Although the concentration of fowlpox virus was on the order of 1-2 log units less than the concentration of vaccinia virus, the minimum concentration of fowlpox virus produced by these cells was approximately 7 × 10^7^ pfu/mL. Vaccinia virus titers were in the 2 × 10^9^ pfu/mL range. The virus used in this work was cell-associated virus that was lysed out of the cells. The virus was then centrifuged to remove any cell debris, which is similar to the manner in which Tanabe and Hotta prepared the variola major used in their work. The purity of the virus used is an important factor. Highly purified virus is more quickly inactivated than crude extracts of virus. The purities of the vaccinia and fowlpox viruses are comparable, as the virus harvesting methods were very similar to the methods described by Tanabe and Hotta. 

Data from this study suggest that fowlpox virus is a suitable decontamination simulant for variola major when using disinfectants at full strength (as they would typically be used). Fowlpox virus and vaccine virus are both inactivated within 1 minute when using the following disinfectants: 70% ethanol, 50% isopropyl alcohol, 0.5% sodium hypochlorite, 30% formaldehyde, 10% benzalkonium chloride, a mixture of 6.67% cetyltrimethylammonium chloride and 3.33% benzalkonium chloride, and a mixture of 1.75% iodine and 10% polyethyleneglycol nonylphenyl ether. 

Viral inactivation with minimal concentrations of the same chemicals was also examined in the present study, for the purpose of determining the inactivation kinetics of both vaccinia and fowlpox. To first principles, the inactivation curves for each virus and chemical appear similar; however mathematical analysis of the data reveals distinct differences for vaccinia and fowlpox viruses treated with 0.05% iodine and 0.3% polyethyleneglycol nonylphenyl ether and 40% ethanol.

The reaction orders described in this study are both first- and second-order decay reactions. First-order reactions were observed with vaccinia and fowlpox viruses treated with 0.1% iodine and 5.7% polyethyleneglycol nonylphenyl ether, 0.025% and 0.05% sodium hypochlorite, and 0.1% cetyltrimethylammonium chloride and 0.05% benzalkonium chloride. Vaccinia virus treated with 40% ethanol also showed a first-order reaction, while a second-order reaction was observed when the virus was treated with 0.05% iodine and 0.3% polyethyleneglycol nonylphenyl ether. Fowlpox virus exhibited second-order reactions when treated with 0.05% iodine and 0.3% polyethyleneglycol nonylphenyl ether and 40% ethanol. 

The first-order decay reaction is typically expected when observing viral kinetics. However, further analysis of the unique properties of poxviruses suggests possible reasons for the observed second-order reaction of fowlpox virus treated with 0.05% iodine and 0.3% polyethyleneglycol nonylphenyl ether and 40% ethanol and the vaccinia virus treated with 0.05% iodine and 0.3% polyethyleneglycol nonylphenyl ether. First, vertebrate poxviruses have the unique ability to nongenetically reactivate. In nongenetic reactivation, an infectious poxvirus particle is able to reactivate a second inactivated poxvirus particle [1]. Secondly, poxviruses (like many other viruses) tend to aggregate in solutions. The aggregated viruses that cannot be neutralized by antibodies are called the persistent fraction or nonneutralizable fraction [10]. Studies have shown that vaccinia virus treated with nitrogen mustard bis (*β*-chloroethyl) methylamine (nitrogen mustard), a chemical known for virus inactivation, can survive treatment with this agent when the particles are aggregated [4]. In fact, Jolik showed that rabbitpox virus treated with nitrogen mustard is still able to act as an “uncoating” agent for other viruses that have been inactivated, but still genetically intact, which is the phenomenon described above for nongenetic reactivation [11]. Based on the data and published research, it could be concluded that fowlpox possibly undergoes greater aggregation than vaccinia virus in both 0.05% iodine and 0.3% polyethyleneglycol nonylphenyl ether and 40% ethanol, resulting in nongenetic reactivation and therefore, a second-order reaction. Thus, the second-order reaction might be due to the fact that fowlpox virus particles in the center of a viral aggregate might be protected from the disinfectant and have the ability to reactive the genetically intact, but inactivated fowlpox virus particles.

Kinetic analysis further elucidated the inactivation similarities and differences between vaccinia and fowlpox viruses. The rate constant, *k*, is calculated from the starting concentration of virus and the concentration of virus at time, *t*. The *k* values differed by no more than 0.7 1/s for the vaccinia and fowlpox viruses treated with 0.1% iodine and 5.7% polyethyleneglycol nonylphenyl ether, 0.025% sodium hypochlorite, 0.05% sodium hypochlorite, and 0.1% cetyltrimethylammonium chloride and 0.05% benzalkonium chloride. However, the vaccinia and fowlpox viruses treated with 0.05% iodine and 0.3% polyethyleneglycol nonylphenyl ether and 40% ethanol show large differences in *k* values. These observed differences could be due to the fact that when treated with 40% ethanol, vaccinia undergoes a first-order reaction and fowlpox undergoes a second-order reaction. When treated with 0.05% iodine and 0.3% polyethyleneglycol nonylphenyl ether, the data suggests that fowlpox virus is more stable in that disinfectant.

Although the *k* value is an important variable for comparison, for the purpose of this work, the virus half-life (*t*_1/2_) value is a better indicator of simulant suitability. The purpose of this work is to determine if fowlpox is a suitable decontamination simulant for variola minor, thus the amount of time the virus will survive is more important than the rate of the reaction. Based on the data presented in Table 1, we can conclude that fowlpox is a suitable decontamination simulant for variola major when using the following disinfectants at concentrations used for inactivation kinetics: 0.1% iodine and 5.7% polyethyleneglycol nonylphenyl ether, 0.025% sodium hypochlorite, 0.05% sodium hypochlorite and 0.1% cetyltrimethylammonium chloride and 0.05% benzalkonium chloride. The similar virus half-life and reaction orders indicate that fowlpox and vaccinia virus undergo similar reactions at similar times. However, fowlpox is not a suitable decontamination simulant for variola major when using 0.05% iodine and 0.3% polyethyleneglycol nonylphenyl ether and 40% ethanol. The differences in reaction order for the two viruses for 40% ethanol are important factors. Additionally, vaccinia is two times more stable in that disinfectant than fowlpox. Fowlpox virus is 20 times more stable than vaccinia virus in 0.05% iodine and 0.3% polyethyleneglycol nonylphenyl ether. 

The studies presented in this paper suggest that fowlpox is a suitable decontamination simulant for variola major when using the following full strength disinfectants: 70% ethanol, 50% isopropyl alcohol, 0.5% sodium hypochlorite, 30% formaldehyde, 10% benzalkonium chloride, a mixture of 6.67% cetyltrimethylammonium chloride and 3.33% benzalkonium chloride and a mixture of 1.75% iodine and 10% polyethyleneglycol nonylphenyl ether. Fowlpox virus is also a suitable inactivation kinetics simulant when treated with the following chemicals: 0.1% iodine and 5.7% polyethyleneglycol nonylphenyl ether, 0.025% sodium hypochlorite, 0.05% sodium hypochlorite and 0.1% cetyltrimethylammonium chloride and 0.05% benzalkonium chloride. Fowlpox virus is not a suitable inactivation kinetics simulant for variola major when treated with the following chemicals: 0.05% iodine and 0.3% polyethyleneglycol nonylphenyl ether and 40% ethanol. Knowing the genetic and decontamination similarities of variola major and vaccinia from previous studies, fowlpox virus has been shown to be a suitable variola major simulant through identical decontamination results using vaccinia virus as the common link between the studies.

Like the decontamination studies, vaccinia virus served as the common link between variola virus and fowlpox virus for the thermal stability studies. Hahon and Kozikowski [8] determined the Δ*H* and Δ*S* of variola major virus in various buffers at temperatures from 40°C to 55°C. Previous studies with vaccinia virus demonstrated that vaccinia virus has a range of Δ*H* values from 20,000 to 90,000 calories per mole [12]. Variola major virus has a Δ*H* values in the range described by Kaplan [12]. The calculated Δ*H* values for the vaccinia virus and fowlpox virus described in this paper fall into the range described Kaplan, with the exception of vaccinia virus heated in brain heart infusion broth. The difference in Δ*H* values in this paper with vaccinia virus in brain heart infusion broth might be due to the decreased virus aggregation, which might have affected the inactivation and plaque counts. Hahon and Kozikowski [8] did not mention decreased aggregation in the variola major virus heated in brain heart infusion broth; however, their variola major sample was stored in brain heart infusion broth, while the viruses used in this work were stored in DMEM containing 10% FBS. The Δ*H* values calculated for vaccinia virus in 0.85% saline, pH 4.5, PBS, pH 7.4, and 10% skim milk were in the range described for both vaccinia [12] and variola major virus [8]. The Δ*H* values calculated for fowlpox virus in 0.85% saline, pH 4.5, PBS, pH 7.4, 10% skim milk, and brain heart infusion broth were in the range described for both vaccinia (Kaplan) and variola major virus [8]. Hahon and Kozikowski [8] also stated that “it is common to find values of Δ*S* of the order of 10 to 100 calories per mol per degree” for the denaturation of proteins. The Δ*S* values described in this work for both vaccinia and fowlpox viruses fall well within the 10–100 calories per mol per degree Celsius range. 

These studies suggest that fowlpox can serve as a thermal stability simulant for variola major in 0.85% saline, pH 4.5, PBS, pH 7.4, 10% skim milk, and heart infusion broth at temperatures ranging from 40°C to 55°C. At temperatures above, 55°C, fowlpox does not appear to be as stable as vaccinia virus. As the storage conditions studies demonstrate, even when stored at −80°C, fowlpox quickly denatures when heated at 56°C when compared to vaccinia virus.

## Figures and Tables

**Table 1:** The effectiveness of disinfectants on vaccinia virus. Table 1 describes the number of plates (based on triplicate assays) with any wells showing cytopathic effects or plaques.

Chemical	Positive control	1 minute treatment	3 minute treatment	5 minute treatment	10 minute treatment
0.05% iodine/0.3% polyethyleneglycol nonylphenyl ether	3/3	0/3	0/3	0/3	0/3

0.1% iodine/5.7% polyethyleneglycol nonylphenyl ether	3/3	0/3	0/3	0/3	0/3

0.025% sodium hypochlorite	3/3	0/3	0/3	0/3	0/3

0.05% sodium hypochlorite	3/3	0/3	0/3	0/3	0/3

0.1% cetyltrimethylammonium chloride/0.05% benzalkonium chloride	3/3	0/3	0/3	0/3	0/3

40% ethanol	3/3	0/3	0/3	0/3	0/3

**Table 2:** The effectiveness of disinfectants on fowlpox virus. Table 1 describes the number of plates (based on triplicate assays) with any wells showing cytopathic effects or plaques.

Chemical	Positive control	1 minute treatment	3 minute treatment	5 minute treatment	10 minute treatment
0.05% iodine/0.3% polyethyleneglycol nonylphenyl ether	3/3	0/3	0/3	0/3	0/3

0.1% iodine/5.7% polyethyleneglycol nonylphenyl ether	3/3	0/3	0/3	0/3	0/3

0.025% sodium hypochlorite	3/3	0/3	0/3	0/3	0/3

0.05% sodium hypochlorite	3/3	0/3	0/3	0/3	0/3

0.1% cetyltrimethylammonium chloride/0.05% benzalkonium chloride	3/3	0/3	0/3	0/3	0/3

40% ethanol	3/3	0/3	0/3	0/3	0/3

**Table 3:** Reaction orders, rates, and virus half-life for vaccinia and fowlpox virus following treatment with low concentrations of four chemicals.

Chemical	Reaction order for vaccinia virus	Reaction order for fowlpox virus	*k* for vaccinia virus*	*k* for fowlpox virus*	*t*_1/2_ for vaccinia virus (s)	*t*_1/2_ for fowlpox virus (s)
0.05% iodine/0.3% polyethyleneglycol nonylphenyl ether	Second	Second	1.63 × 10^−6^	2.43 × 10^−9^	1.04 × 10^−4^	25.40

0.1% iodine/5.7% polyethyleneglycol nonylphenyl ether	First	First	0.128	0.0976	5.4	7.07

0.025% sodium hypochlorite	First	First	0.125	0.082	5.52	8.41

0.05% sodium hypochlorite	First	First	0.125	0.0841	5.53	8.2

0.1% cetyltrimethylammonium chloride/0.05% benzalkonium chloride	First	First	0.118	0.0922	5.84	7.48

40% ethanol	First	Second	0.0168	1.90 × 10^−8^	41.06	1.97

*The units of *k* for first-order reactions are 1/s. The units of *k* for second-order reactions are 1/(M∗s).

**Table 4:** Δ*H* (heat of activation) of vaccinia and fowlpox viruses in various solutions. Values are reported in calories per mole.

Chemical	Δ*H* for vaccinia	Δ*H* for fowlpox
0.85% saline, pH 4.5	44138	53481
PBS, pH 7.4	34239	35868
10% skim milk	53299	22378
Heart infusion broth	95204	65229

**Table 5:** Δ*S* (entropy of activation) of vaccinia virus in various solutions at 40, 45, 50 and 55°C. Values reported in Calories per mol per degree Celsius.

Chemical	40°C	45°C	50°C	55°C
0.85% saline, pH 4.5	67.61	67.46	65.09	65.85
PBS, pH 7.4	69.08	64.33	65.67	63.17
10% skim milk	70.04	67.85	63.83	62.82
Heart infusion broth	64.86	66.01	66.67	63.12

**Table 6:** Δ*S* (Entropy of Activation) of Fowlpox virus in various solutions at 40, 45, 50 and 55°C. Values reported in Calories per mol per degree Celsius.

Chemical	40°C	45°C	50°C	55°C
0.85% Saline, pH 4.5	71.70	69.36	66.85	63.93
PBS, pH 7.4	68.34	68.92	69.11	62.45
10% Skim Milk	67.74	68.53	65.65	65.12
Heart Infusion Broth	73.95	70.50	70.07	63.55
